# The complete chloroplast genome sequence of *Camellia yangii* D.Wei Zhao (Theaceae), a rare tea plant germplasm

**DOI:** 10.1080/23802359.2026.2680730

**Published:** 2026-06-03

**Authors:** Hui-Qun Deng, Xian-Jun Liao, Shi-Xiong Yang, Xiang-Qin Yu, Zhu-Sheng Liu

**Affiliations:** ^a^ Guangxi Key Laboratory of Tea Plant Germplasm Innovation and Resource Utilization, Guangxi Institute of Tea Science, Guilin, China; ^b^ Guilin Field Scientific Observation and Research Station for Crop Germplasm Resources, Ministry of Agriculture and Rural Affairs, Guilin, China; ^c^ Guangxi Field Scientific Observation and Research Station for Tea Resources, Guilin, China; ^d^ Key Laboratory for Plant Diversity and Biogeography of East Asia, Kunming Institute of Botany, Chinese Academy of Sciences, Kunming, China

**Keywords:** *Camellia yangii*, chloroplast genome, Illumina sequencing, phylogenetic analysis

## Abstract

*Camellia yangii* D.Wei Zhao (Theaceae) is a recently reported new tea plant species from Yunnan, China. In this study, we report and characterize the complete chloroplast (cp) genome of *C. yangii*. The cp genome of *C. yangii* is 156,566 bp in length, comprising an 86,233 bp large single-copy region, an 18,255 bp small single-copy region, and a pair of 26,039 bp inverted repeat regions. The genome contains 129 genes, including 36 tRNA, eight rRNA, and 85 protein-coding genes. Phylogenetic analysis indicated *C. yangii* shows closest phylogenetic relationships with *C. makuanica*, *C. kwangsiensis*, *C. crassicoluma*, and *C. taliensis.*

## Introduction

*Camellia* is the largest genus in Theaceae, with about 120 species distributed in East and Southeast Asia (Ming 2000; Ming and Bartholomew 2007). The center of species diversity for the genus is the Southern areas of Yangtze River of China (Ming and Zhang 1996). Tea plants (*Camellia* sect. *Thea*), i.e. tea and its relatives, can be used as beverage sources, their wild populations are the most critical germplasm resources of current cultivars of tea and gained much attention recently (Jiang et al. 2024; Tong et al. 2024; Zhao 2024). *Camellia yangii* D.Wei Zhao 2025 is one of these species and was recently reported as new taxon (with three sepals, purplish red and densely pubescent terminal bud), it represents a rare germplasm resource of tea plants (Zhao 2025). It grows in tropical evergreen montane forest in Malipo County in Yunnan province. Its fertility rate is very low, only one population and less than 10 individuals are known (Zhao 2025). Although the taxonomy of *C. yangii* was well described, no genome sequences of this species are published. In this study, we present the complete chloroplast (cp) genome sequence of *C. yangii* to determine its systematic position in *Camellia* and to contribute to the future phylogenetic and taxonomic studies of the genus.

## Materials and methods

**Figure 1.**
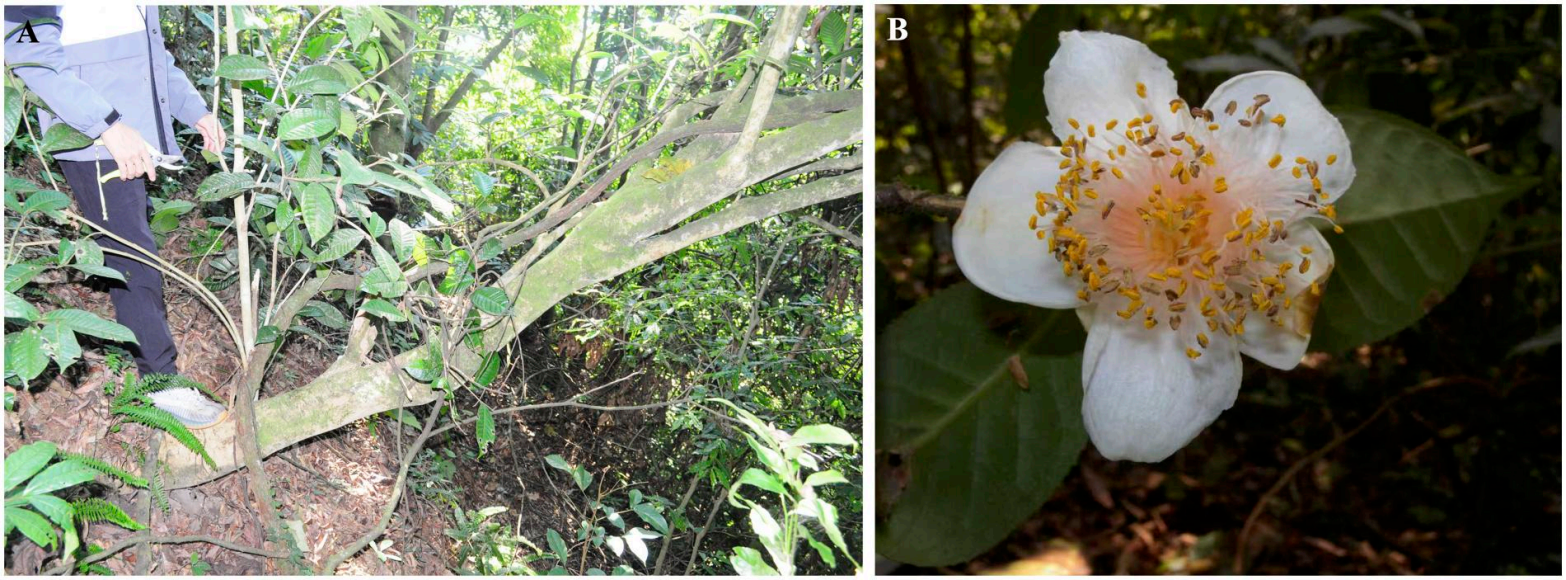
Reference images of *Camellia yangii.* The habitat of *C. yangii* is 5–8 m tall shrub or tree growing in the evergreen montane forest in Malipo county of Yunnan province in China (A). The characteristic morphological feature of *C. yangii* is the three sepals, purplish red and densely pubescent terminal bud (B). Photographs were taken by the author of this article (Shi-Xiong Yang) in Malipo county of Yunnan province in China on 25 December 2023.

Fresh leaves of *C. yangii* were collected from its only known natural population in Malipo county from southern Yunnan, China (23°07′18ʺ N, 104°41′45ʺ E). This study aims to generate vital genetic data for the conservation of this endangered species. The voucher specimen (YangSX 7312) was deposited at the Herbarium at Kunming Institute of Botany (KUN, http://www.kun.ac.cn, Jing-Hua Wang, wangjh@mail.kib.ac.cn), Chinese Academy of Sciences, and it was identified as *C. yangii* by one of the author Shi-Xiong Yang (Figure 1).

Total genomic DNA was extracted using a modified hexadecyltrimethylammonium bromide (CTAB) method (Doyle and Doyle 1987). The 150 bp pair-end reads were generated using the Illumina Hi-Seq 2500 platform (San Diego, CA). Clean reads was de novo assembled using a recent developed python package GetOrganelle (Jin et al. 2020), followed by using Bandage 0.8.1 (Wick et al. 2015) to assess the completeness of the assembly. The coverage depth of the genome was determined using SAMtools v1.6 (Li et al. 2009), and the sequencing depth and coverage map were drawn using ggplot2 (Ito and Murphy 2013) in R. The online platforms OGDRAW v1.3.1 (Greiner et al. 2019) were used to generate a circular genome map. PGA (Qu et al. 2019) was employed to annotate the cp genome using the default settings.

Based on Chang’s classification system of *Camellia* (Chang and Ren 1998), we selected 120 samples (mainly with accessions beginning with NC from GenBank) within the genus to construct a maximum-likelihood (ML) tree. To more clearly illustrate the relationships among the new species and closely related species, we selected 49 *Camellia* samples. Representative species from almost all sections of the genus with sequencing data were integrated, with 19 species belonging to *Camellia* sect. *Thea.* Multiple accessions of *Camellia taliensis* were used.

We used jModelTest v0.11 (Posada 2008) to select the best-fitting nucleotide substitution models. The phylogenetic tree was constructed based on the complete cp genome sequences of *Camellia yangii* and another 48 *Camellia* species, using *Polyspora axillaris* and *Pyrenaria oblongicarpa* as outgroups. Phylogenetic analysis was performed using IQTREE version 2.4.0 (Minh et al. 2020) under the best-fit model and node support was assessed with 5000 bootstrap replicates.

## Results

**Figure 2.**
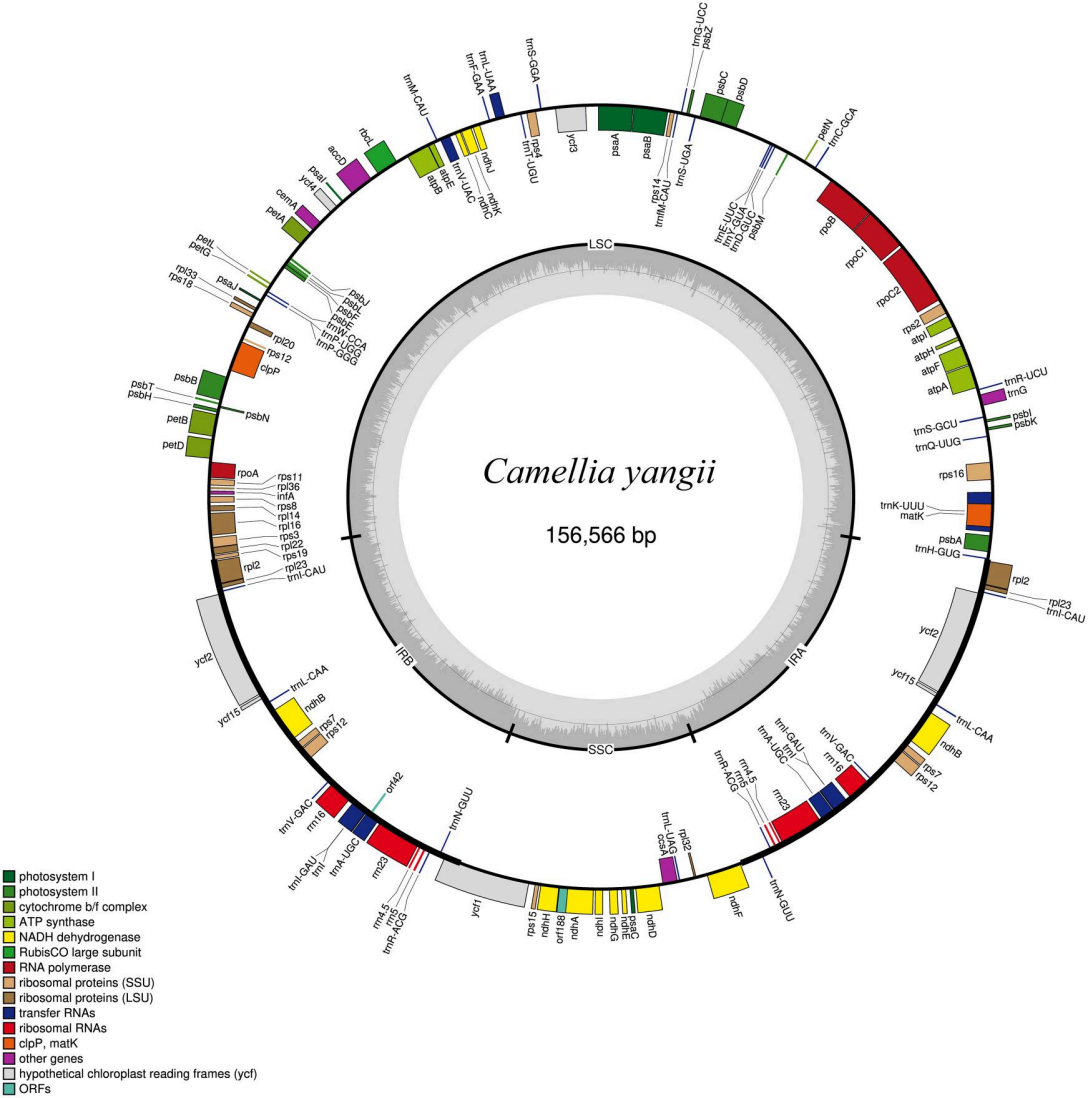
Map of the chloroplast genome of *Camellia yangii* (GenBank ID: PX512987). Conserved genes are shown as colored boxes; those on the inner circle are transcribed clockwise, and those on the outer circle counterclockwise. The innermost ring displays the plastid genome’s GC content as gray bar graphs.

The average sequencing depth was ×962.1, with a minimum coverage of ×297 and a maximum coverage of ×7999. The absence of uncovered regions indicated a high degree of assembly completeness and reliability (Supplementary Figure 1). The cp genome of *C. yangii* (GenBank accession number is PX512987) is 156,566 bp in length, exhibiting a complete and circular structure (Figure 2). The GC content of the genome is 37.3%. This genome includes an 86,233 bp large single-copy region, an 18,255 bp small single-copy region, and two 26,039 bp inverted repeat regions. In total, the genome contains 129 genes, with 36 tRNA, eight rRNA, and 85 protein-coding genes. The 11 cis-spliced genes and one trans-spliced gene (*rps12*) were verified to be corrected and annotated with multiple sequence alignment (Supplementary Figures 2 and 3).

**Figure 3.**
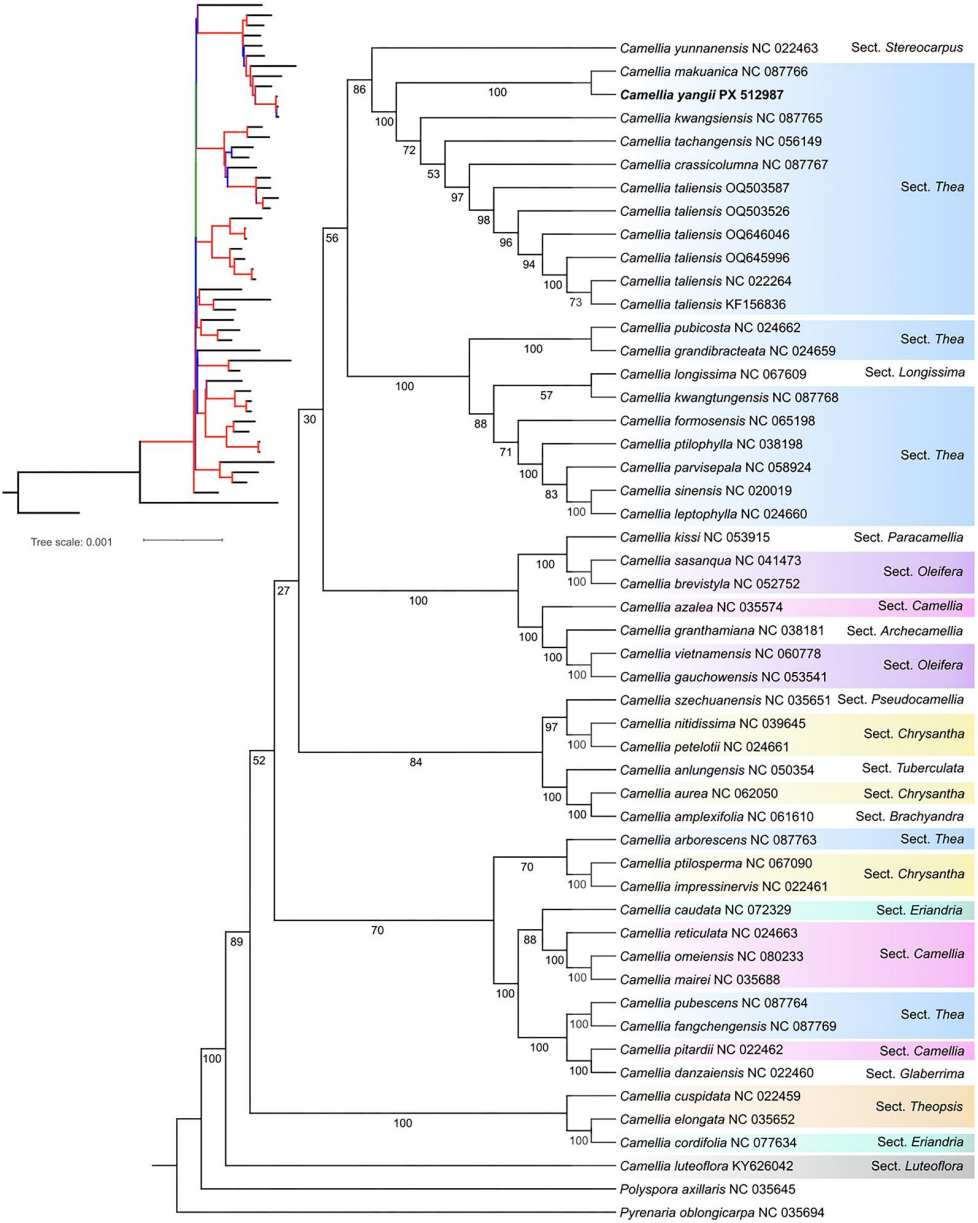
Maximum-likelihood tree of Theaceae based on 51 complete chloroplast genome sequences, including *Camellia yangii* (GenBank ID: PX512987) sequenced in this study. The bootstrap support values are shown beside the nodes. The upper left panel shows ML trees with branch lengths. Red indicates BS = 100%, blue represents 50–99%, and green denotes 27–49%. Scale bar refers to a phylogenetic distance of 0.001 nucleotide substitutions per site. Two representative taxa of Theaceae (*Polyspora axillaris*, NC035645; *Pyrenaria oblongicarpa*, NC035694) were used as outgroups. The GenBank accession numbers for the sequences used are as follows: NC022459 (Yang et al. 2013), NC035625(Yu et al. 2017), NC077634, NC087763 (Jiang et al. 2024), NC067090 (Tang et al. 2024), NC022461 (Yang et al. 2013), NC072329 (Hu and Liu 2024), NC024663 (Huang et al. 2014), NC080233, NC035688 (Yu et al. 2017), NC087764 (Jiang et al. 2024), NC087769 (Jiang et al. 2024), NC022462 (Yang et al. 2013), NC022460 (Yang et al. 2013), NC035651 (Yu et al. 2017), NC039645 (Liu et al. 2018), NC024661 (Huang et al. 2014), NC050354 (Zhu et al. 2020), NC062050 (Tang et al. 2024), NC061610, NC053915 (Cao et al. 2020), NC041473 (Lin et al. 2019), NC052752 (Wang et al. 2020), NC035574 (Lin et al. 2019), NC038181 (Li et al. 2018; Li, Xing, et al. 2018), NC060778, NC053541 (Zhang and Wang 2020), NC024662 (Huang et al. 2014), NC024659 (Huang et al. 2014), NC067609, NC087768 (Jiang et al. 2024), NC065198 (Wang et al. 2024), NC038198 (Li et al. 2018; Li, Xing, et al. 2018), NC058924, NC020019 (Liang et al. 2023), NC024660 (Huang et al. 2014), NC022463 (Yang et al. 2013), NC087766 (Jiang et al. 2024), NC087765 (Jiang et al. 2024), NC056149 (Hao et al. 2019), NC087767 (Jiang et al. 2024), OQ503587 (Shen et al. 2025), OQ503526 (Shen et al. 2025), OQ646046 (Jiang et al. 2024),OQ645996 (Jiang et al. 2024), NC022264 (Yang et al. 2013), KF156836 (Yang et al. 2013), and KY626042 (Wang et al. 2017).

The best-fitting nucleotide model selected by jModeltest was TVM + I + G for the dataset. The phylogenetic tree with 120 samples of *Camellia* yielded moderate resolution at the shallow nodes but low resolution in the deep nodes (Supplementary Figure 4). The phylogenetic tree was shown in Figure 3; seven main clades were identified. *Camellia* sect. *Thea* grouped into four different clades. One clade included *C. yangii*, *C. makuanica*, *C. kwangsiensis*, *C. tachangensis*, *C. crassicolumna*, and *C. taliensis* from *C.* sect. *Thea* (BS = 100%). Another clade contains eight species of *Camellia* sect. *Thea*, it includes *C. leptophylla*, *C. sinensis*, *C. parvisepala*, *C. ptilophylla*, *C. formosensis*, *C. kwangtungensis*, *C. grandibracteata*, and *C. pubicosta* (BS = 100%). The third clade contains two species from *C.* sect. *Thea*, i.e. *C. pubescens* and *C. fangchengensis* (BS = 100%). The fourth clade contains only one species of *Camellia* sect. *Thea*, *C. arborescens*, which is closely related to *C. ptilosperma* and *C. impressinervis* (BS = 70%).

## Discussion and conclusions

This study presents the first assembly and annotation of the complete cp genome of *C. yangii*. The plastome structure aligns with typical angiosperm cp genomes, characterized by a quadripartite organization and moderate GC content (37.3%). The presence of 129 genes, including 11 cis-spliced genes and the fragmented *rps12* gene is comparable with other *Camellia* species, such as *C. mingii* (Zhang et al. 2019), *C. tachangensis* (Hao et al. 2019), and *Camellia sinensis* (Liang et al. 2023). The data will enrich the genomic resources available for *Camellia* and providing insights into the phylogenetic relationships within *Camellia* sect. *Thea.*

Our phylogenetic analysis revealed that *C. yangii*, *C. makuanica*, *C. kwangsiensis*, *C. crassicoluma*, and *C. taliensis* (all mainly distributed in Yunnan province) (Ming and Bartholomew 2007; Zhao 2024) grouped into a monophyletic clade (BS = 100%), consolidating its placement within the *Camellia* sect. *Thea.* This close affinity suggests shared evolutionary adaptations potentially linked to ecological niches in Southwestern China (Yu et al. 2017). These results are consistent with the placement based on morphology, highlighting similarities in sepal and terminal bud (Zhao 2024). Given the moderate resolution of our plastid phylogeny, additional nuclear genomic data (Shen et al. 2025) will be crucial to further assess the phylogenetic position of this endangered species and clarify its taxonomic boundaries within *Camellia* sect. *Thea*.

In conclusion, the newly sequenced cp genome of *C. yangii* is a valuable resource for future phylogenetic studies of *Camellia*. Furthermore, this genomic resource could aid conservation efforts by facilitating population genetic studies, species identification, and assessment of genetic diversity in wild populations of this endangered tea plant species.

## Supplementary Material

Supplemental material_20260210.docx

## Data Availability

The data that support the findings of this study are openly available in GenBank of NCBI at https://www.ncbi.nlm.nih.gov/nuccore/PX512987, reference number PX512987. The associated BioProject number is PRJCA049009 in the National Genomics Data Center of China at https://ngdc.cncb.ac.cn/search/specific?-db=bioproject&q=PRJCA049009.

## References

[CIT0001] Cao L, Li J, Fan Z, Yin H, Li X. 2020. Characterization and phylogenetic significance of the complete chloroplast genome of *Camellia kissii*, an economic crop for producing oil. Mitochondrial DNA B Resour. 5(1):362–363. 10.1080/23802359.2019.1703569 33366557 PMC7748572

[CIT0002] Chang HT, Ren SX. 1998. Theaceae. In: Wu, C.Y., editor. Flora Reipublicae Popularis Sinicae. Science Press.

[CIT0004] Doyle JJ, Doyle JL. 1987. A rapid DNA isolation procedure for small quantities of fresh leaf tissue. Phytochem Bull. 19(1):11–15.

[CIT0005] Greiner S, Lehwark P, Bock R. 2019. OrganellarGenomeDRAW (OGDRAW) version 1.3.1: expanded toolkit for the graphical visualization of organellar genomes. Nucleic Acids Res. 47(W1):W59–W64. 10.1093/nar/gkz238 30949694 PMC6602502

[CIT0006] Hao WJ, Ma JQ, Ma C, Jin J, Chen L. 2019. The complete chloroplast genome sequence of *Camellia tachangensis* F. C. Zhang (Theaceae). Mitochondrial DNA B Resour. 4(2):3344–3345. 10.1080/23802359.2019.1673247 33365985 PMC7707387

[CIT0007] Hu Y, Liu BB. 2024. Analyses on chloroplast genome characteristics and phylogeny of *Camellia cordifolia*. J Plant Resour Environ. 33(3):1–13.

[CIT0008] Huang H, Shi C, Liu Y, Mao S-Y, Gao L-Z. 2014. Thirteen *Camellia* chloroplast genome sequences determined by high-throughput sequencing: genome structure and phylogenetic relationships. BMC Evol Biol. 14:151. 25001059 10.1186/1471-2148-14-151 PMC4105164

[CIT0009] Ito K, Murphy D. 2013. Application of ggplot2 to pharmacometric graphics. CPT Pharmacometr Syst Pharmacol. 2(10):e79. 10.1038/psp.2013.56 PMC3817376 24132163

[CIT0010] Jiang YZ et al. 2024. Species delimitation of tea plants (*Camellia* sect. *Thea*) based on super-barcodes. BMC Plant Biol. 24(1):181. 10.1186/s12870-024-04882-3 38468197 PMC10926627

[CIT0011] Jin JJ et al. 2020. GetOrganelle: a fast and versatile toolkit for accurate de novo assembly of organelle genomes. Genome Biol. 21(1):241. 10.1186/s13059-020-02154-5 32912315 PMC7488116

[CIT0012] Li H et al. 2009. The sequence alignment/map format and SAMtools. Bioinformatics. 25(16):2078–2079. 10.1093/bioinformatics/btp352 19505943 PMC2723002

[CIT0014] Li WX et al. 2018. Characterization of the complete chloroplast genome of *Camellia granthamiana* (Theaceae), a vulnerable species endemic to China. Mitochondrial DNA B Resour. 3(2):1139–1140. 10.1080/23802359.2018.1521310 33474444 PMC7800109

[CIT0015] Li WX, Xing F, Ng WL, Zhou YB, Shi XG. 2018. The complete chloroplast genome sequence of *Camellia ptilophylla* (Theaceae): a natural caffeine-free tea plant endemic to China. Mitochondrial DNA B Resour. 3(1):426–427. 10.1080/23802359.2018.1457996 33474191 PMC7799551

[CIT0016] Liang YN et al. 2023. The complete chloroplast genome and phylogenomic analysis of *Camellia sinensis* var. *sinensis* cultivar ‘Liupao’, a landrace from Guangxi, China. Mitochondrial DNA B Resour. 8(8):921–926. 10.1080/23802359.2023.2250072 37645477 PMC10461518

[CIT0017] Lin HY et al. 2019. Phylogenomic conflict resulting from ancient introgression following species diversification in *Stewartia* s.l. (Theaceae). Mol Phylogenet Evol. 135:1–11. 10.1016/j.ympev.2019.02.018 30802596

[CIT0018] Lin Y et al. 2019. Characterization of the complete chloroplast genome of *Camellia renshanxiangiae* (Theaceae). Mitochondrial DNA B Resour. 4(1):1490–1491. 10.1080/23802359.2019.1601041

[CIT0019] Liu MM et al. 2018. Characterization of the complete chloroplast genome of the *Camellia nitidissima*, an endangered and medicinally important tree species endemic to Southwest China. Mitochondrial DNA B Resour. 3(2):884–885. 10.1080/23802359.2018.1501304 33474353 PMC7800646

[CIT0020] Ming TL, Zhang WJ. 1996. The evolution and distribution of genus *Camellia*. Acta Bot Yunnan. 18(1):1–13.

[CIT0021] Ming TL. 2000. Monograph of the genus *Camellia*. Yunnan Science and Technology Press.

[CIT257525] Ming TL, Bartholomew B. 2007. Theaceae. In: Wu CY, Raven, PH, Hong DY, editors. *Flora of China.* (Vol. 12). Science Press, Beijing and Missouri Botanical Garden Press.

[CIT0022] Minh BQ et al. 2020. IQ-TREE 2: new models and efficient methods for phylogenetic inference in the genomic era. Mol Biol Evol. 37(5):1530–1534. 10.1093/molbev/msaa015 32011700 PMC7182206

[CIT0023] Posada D. 2008. jModelTest: phylogenetic model averaging. Mol Biol Evol. 25(7):1253–1256. 10.1093/molbev/msn083 18397919

[CIT0024] Qu XJ, Moore MJ, Li DZ, Yi TS. 2019. PGA: a software package for rapid, accurate, and flexible batch annotation of plastomes. Plant Methods. 15(1):50. 10.1186/s13007-019-0435-7 31139240 PMC6528300

[CIT0025] Shen ZF et al. 2025. Genomic DNA barcodes provide novel insights into species delimitation in the complex *Camellia* sect. *Thea* (Theaceae). BMC Plant Biol. 25(1):570. 10.1186/s12870-025-06612-9 40307692 PMC12044775

[CIT0027] Tang Y et al. 2024. The complete chloroplast genome of *Camellia flava* (Pitard) Sealy, a golden camellia of Vietnam. Mitochondrial DNA B Resour. 9(8):1117–1121. 10.1080/23802359.2024.2392741 39175482 PMC11340231

[CIT0028] Tong W et al. 2024. Genomic variation of 363 diverse tea accessions unveils the genetic diversity, domestication, and structural variations associated with tea adaptation. J Integr Plant Biol. 66(10):2175–2190. 10.1111/jipb.13737 38990113

[CIT0029] Wang G, Luo Y, Hou N, Deng LX. 2017. The complete chloroplast genomes of three rare and endangered Camellias (*Camellia huana*, *C. liberofilamenta* & *C. luteoflora*) endemic to Southwest China. Conserv Genet Resour. 9(4):583–585. 10.1007/s12686-017-0727-z

[CIT0030] Wang LJ et al. 2024. The plastome of *Camellia formosensis* (Theaceae) reconfirms the endemism of Taiwanese wild tea. Taiwan J Forest Sci. 39(3):231–240.

[CIT0031] Wang Y et al. 2020. Characterization of the complete chloroplast genome of *Camellia brevistyla*, an oil-rich and evergreen shrub. Mitochondrial DNA B Resour. 5(1):386–387. 10.1080/23802359.2019.1703607 33366568 PMC7748852

[CIT0032] Wick RR, Schultz MB, Zobel J, Holt KE. 2015. Bandage: interactive visualization of de novo genome assemblies. Bioinformatics. 31(20):3350–3352. 10.1093/bioinformatics/btv383 26099265 PMC4595904

[CIT0034] Yang JB, Yang SX, Li HT, Yang J, Li DZ. 2013. Comparative chloroplast genomes of *Camellia* species. PLOS One. 8(8):e73053. 10.1371/journal.pone.0073053 24009730 PMC3751842

[CIT0035] Yu XQ et al. 2017. Insights into the historical assembly of East Asian subtropical evergreen broadleaved forests revealed by the temporal history of the tea family. New Phytol. 215(3):1235–1248. 10.1111/nph.14683 28695680

[CIT0036] Zhang Q et al. 2019. The complete chloroplast genome sequence of *Camellia mingii* (Theaceae), a critically endangered yellow camellia species endemic to China. Mitochondrial DNA B Resour. 4(1):1338–1340. 10.1080/23802359.2019.1596765

[CIT0037] Zhang Y, Wang G. 2020. The complete chloroplast genome of *Camellia gauchowensis* and its phylogenetic analysis. Mitochondrial DNA B Resour. 5(3):2299–2300. 10.1080/23802359.2020.1772694 33457767 PMC7782028

[CIT0038] Zhao DW. 2024. Botany and taxonomy of tea (*Camellia sinensis*, Theaceae) and its relatives. In: Chen L, Chen JD, editors. The tea plant genome. Springer. p 13–37. 10.1007/978-981-97-0680-8_2

[CIT0039] Zhao DW. 2025. *Camellia yangii* (Theaceae), a new species of tea plants (*Camellia* section *Thea*). PhytoKeys. 257:247–256. 10.3897/phytokeys.257.152000 40636267 PMC12239013

[CIT0040] Zhu Y et al. 2020. Characterization of the complete chloroplast genome of *Camellia anlungensis*. Mitochondrial DNA B Resour. 5(1):873–874. 10.1080/23802359.2020.1716639 33366791 PMC7748807

