# Supplementary material for: The complete chloroplast genome sequence of Camellia yangii D.Wei Zhao (Theaceae), a rare tea plant germplasm
Source: Mitochondrial DNA B Resour. 2026 Jun 3;11(7):822–6. doi: 10.1080/23802359.2026.2680730 (PMC13235223; doi:10.1080/23802359.2026.2680730)
Supplement: Supplemental material_20260210.docx [file TMDN_A_2680730_SM2788.docx]

**Supplemental material**

**
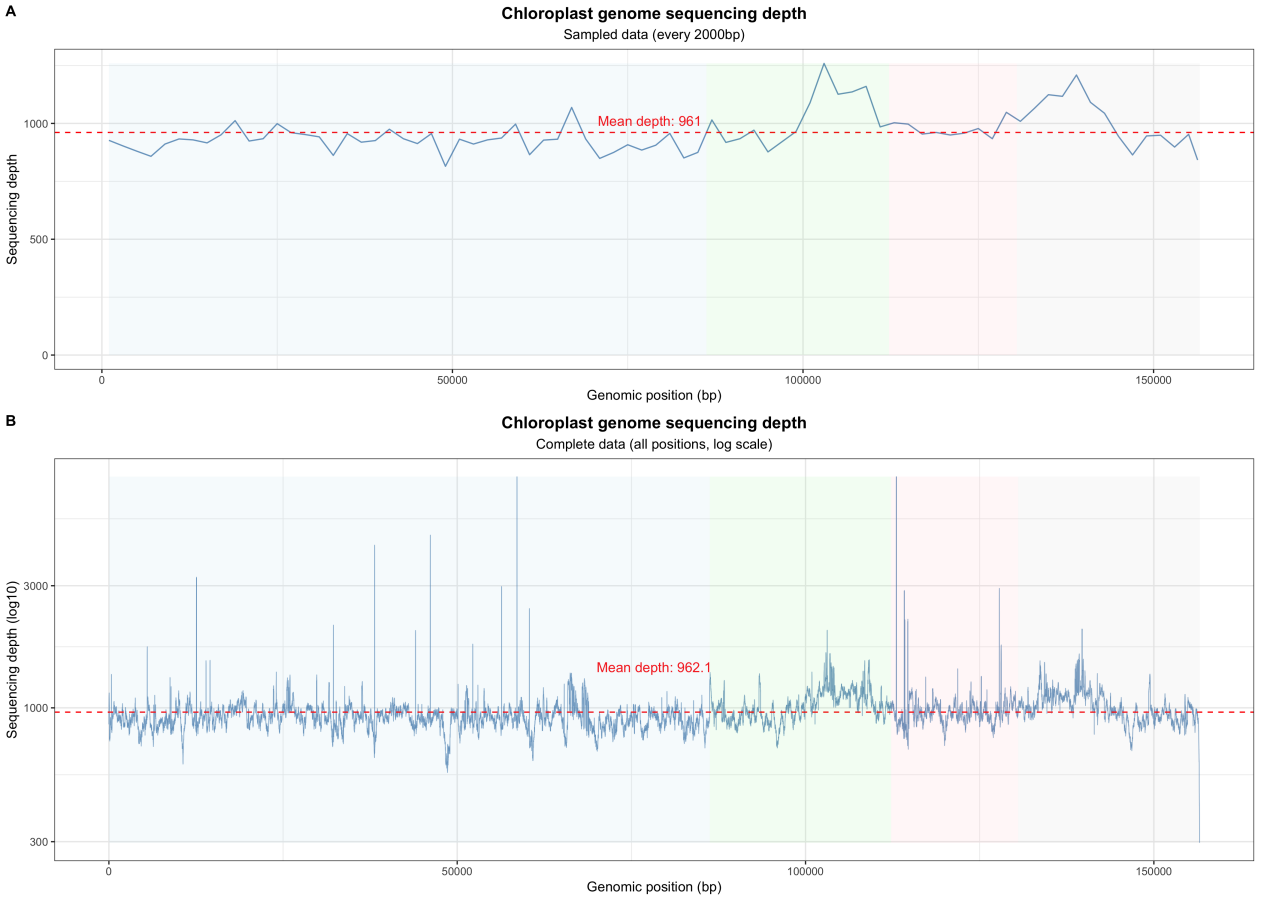
**

Supplementary Figure 1. Coverage depth distribution of the chloroplast genome in *Camellia yangii*. (A) Moving average depth, (window=2,000 bp, step=2,000 bp) with annotated structural domains. (B) Raw depth values with smoothing spline, window=1 bp. (blue, λ=0.1) (LSC,blue; IRb:green; SSC:pink; IRa,gray).

**
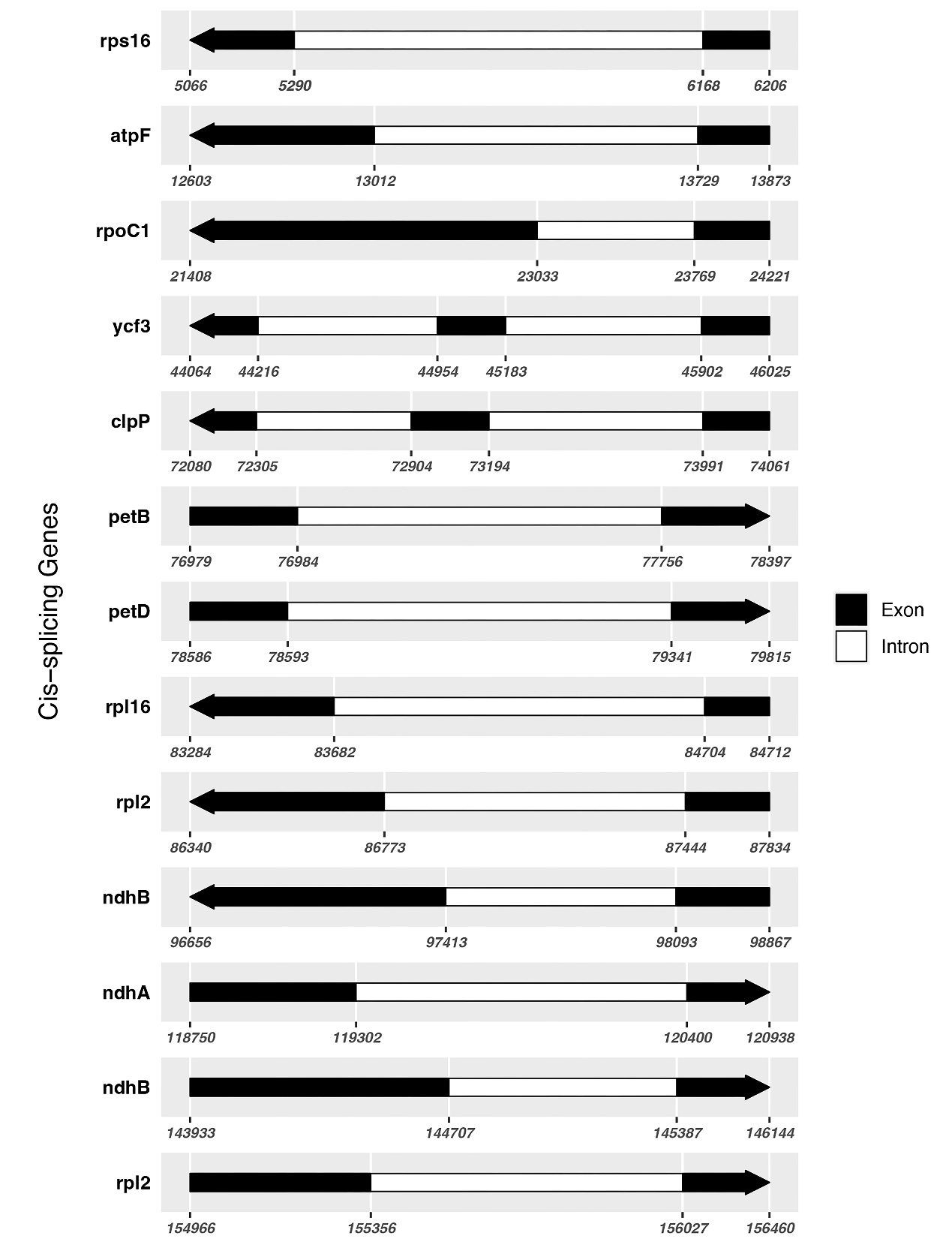
**

Supplementary Figure 2. The cis-splicing genes in the chloroplast genome of *Camellia yangii* are depicted in a schematic map using CPGView. The exons of the cis-splicing genes are represented in black, while the introns are shown in white. An arrow indicates the sense direction of the gene.


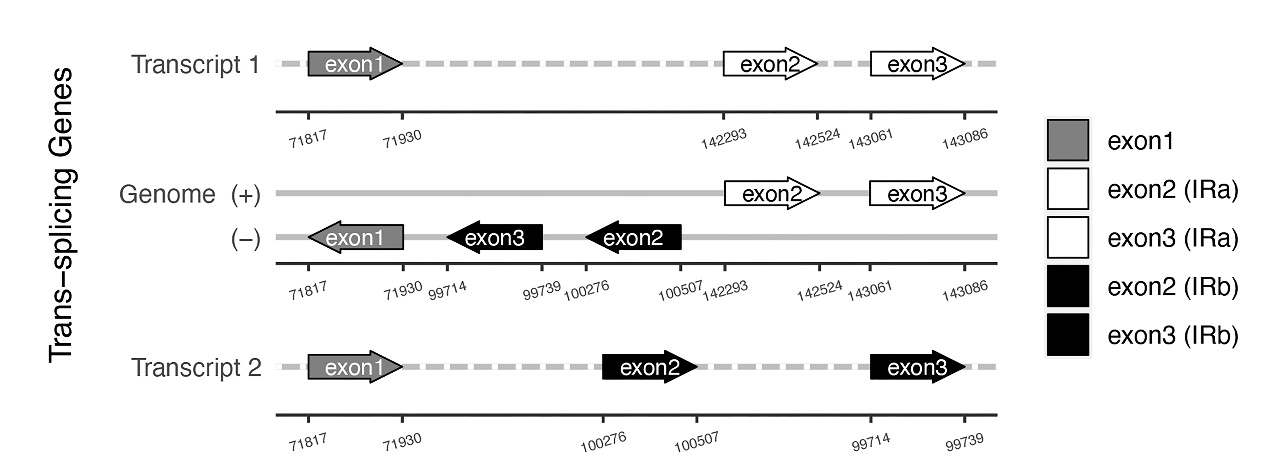


Supplementary Figure 3. Schematic map of the trans-splicing gene *rps12* in the chloroplast genome of *Camellia yangii*. It has three unique exons. Two of them are duplicated as they are located in the IR regions.


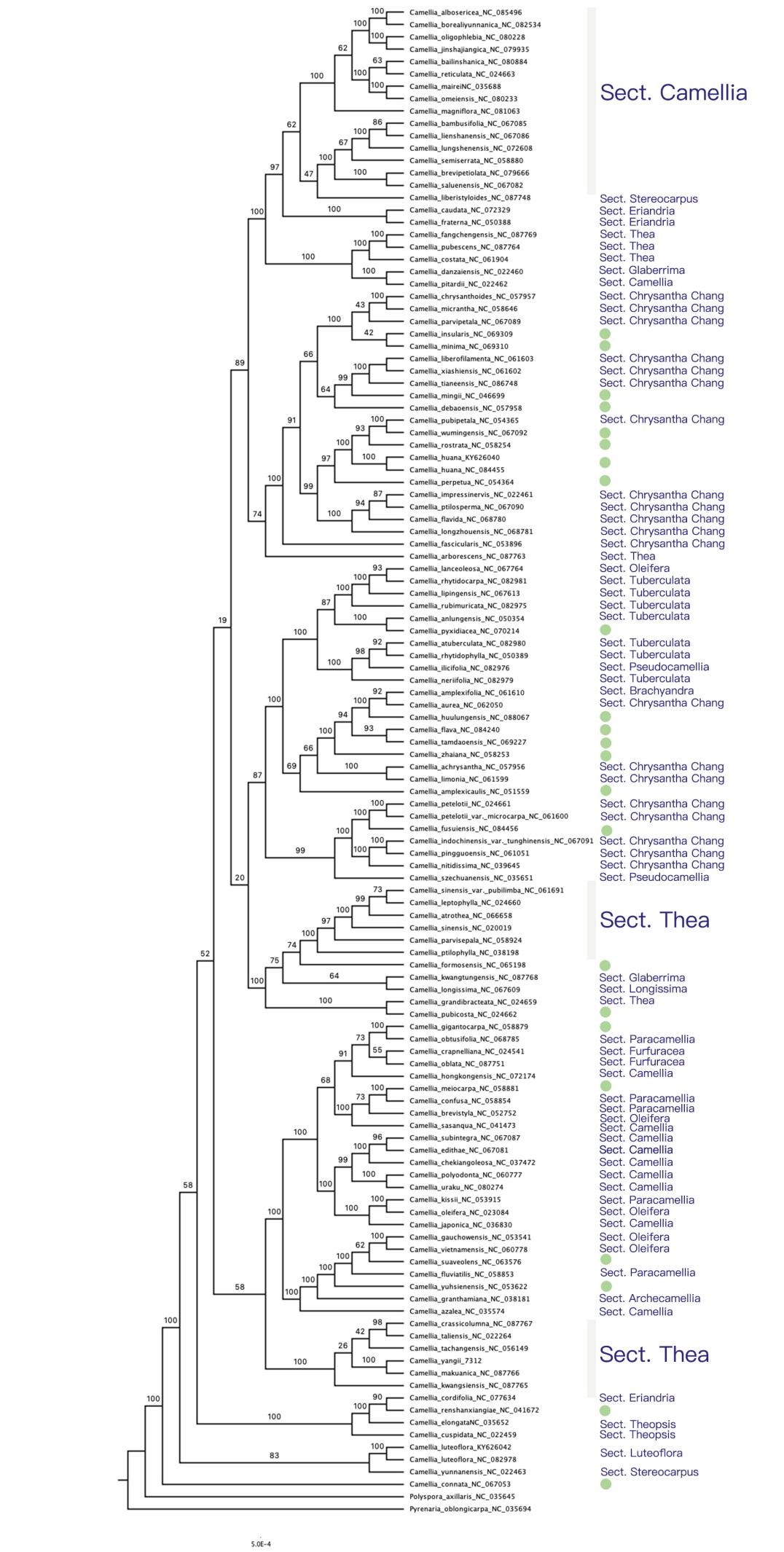


Supplementary Figure 4. Maximum likelihood tree of *Camellia* based on 122 complete chloroplast genome sequences, using *Polyspora axillaris* and *Pyrenaria oblongicarpa* as outgroups. The green point indicates that those species are not included in Chang's classification system of *Camellia*.
